# Unidentified Object in the Mediastinum: A Case Report of Severe Aortic Calcification in a Patient With Rheumatoid Arthritis

**DOI:** 10.7759/cureus.30580

**Published:** 2022-10-22

**Authors:** Qusai T Alitter, Islam S Gadelmoula, Sahar S Abdelmoneim, Fatmaelzahraa Abosbeaa, Sabas Gomez

**Affiliations:** 1 Pulmonary Disease, Larkin Community Hospital, Hialeah, USA; 2 Internal Medicine, Larkin Community Hospital, Hialeah, USA; 3 Internal Medicine/Cardiovascular Medicine, Assiut University Hospital, Assiut, EGY; 4 Internal Medicine, Cairo University, Cairo, EGY; 5 Cardiology, Larkin Community Hospital, South Miami, USA

**Keywords:** inflammatory illness, rheumatology & autoimmune diseases, cardiovascular prevention, vascular calcification, rheumatoid arthriitis, aortic calcification

## Abstract

We present a case of an 80-year-old female with a past medical history of rheumatoid arthritis (RA) who was incidentally found to have severe circumferential thoracic aortic calcification detected on chest X-ray and computed tomography (CT) scan of the chest. This case highlights the chronic inflammatory state and immunological vascular damage as key mechanisms for the accumulation of dystrophic calcification in the blood vessels and soft tissues of patients with autoimmune and inflammatory diseases. It also emphasizes the importance of coordinated multidisciplinary care and management between different specialties including primary care physician (PCP), cardiology, and rheumatology to address all the challenges related to disease control and optimize cardiovascular risk factors in this patient population.

## Introduction

The incidental finding of a thoracic aortic calcification or a porcelain aorta on chest imaging in patients undergoing evaluation for cardiopulmonary disease is not uncommon. Several risk factors and disease processes can precipitate the development of vascular calcification including atherosclerotic risk factors, systemic inflammatory conditions, chronic kidney disease, and mediastinal irradiation [1]. In the porcelain aorta specifically, there is heavy calcification that can take a complete or near complete circumferential pattern in the anterior wall of the ascending aorta and the superior wall of the aortic arch [2]. Vascular calcification is further described based on its histological location which can be within the tunica intima (i.e., atherosclerotic) or within the tunica media (i.e., non-atherosclerotic). The clinical significance of severe aortic calcification and porcelain aorta emerges from the increased risk of periprocedural complications during cardiothoracic surgeries from different surgical techniques including aortic clamping, canulation, autotomy during aortic valve replacement, and/or coronary artery bypass grafting. Detection of asymptomatic coronary artery and aortic calcification in patients with rheumatoid arthritis (RA) has been reported and correlated with disease duration. Recognizing this is important, given the increased risk of cardiovascular disease (CVD) with RA and the beneficial effects of disease-modifying antirheumatic drugs (DMARDs) [3]. Porcelain aorta in RA patients is scarcely reported in the literature [4].

We present a case of an 80-year-old female with respiratory complaints whose chest X-ray showed an incidental “unidentified object” in the mediastinum, which was further revealed to be a circumferential calcification of the thoracic aorta on chest CT scan.

## Case presentation

An 80-year-old Hispanic female with a past medical history of essential hypertension, hypothyroidism, and RA presented to the hospital with a one-week history of runny nose and productive cough. Her home medications included hydrochlorothiazide, lisinopril, methotrexate (15 mg per week), and Vitamin D3. Physical examination was remarkable for subcutaneous nodules on the hands and feet, swan neck deformity of the hands and bilateral inspiratory crackles on lung auscultation. Blood tests and diagnostic workup of the patient are provided in Table 1.

**Table 1:** Blood Tests Blood tests revealed low methotrexate level indicating medications noncompliance and elevated cholesterol and low-density lipoprotein level

Blood Tests and Diagnostic Workup	Result	Reference Range
White Blood Cells	5.6 K/mcL	3.4-11.0 K/mcL
Hemoglobin	10.7 g/dl	12-15 g/dl
Hematocrit	33.5%	35-45%
Mean Corpuscular Volume	88 fL	80-100 fL
Platelets	156 K/mcL	150-450 K/mcL
Blood Urea Nitrogen	15 mg/dl	8-26 mg/dl
Serum Creatinine	0.94 mg/dl	0.8-1.4 mg/dl
Lactic Acid	0.8 nmol/L	0.7-2 nmol/L
C-Reactive Protein	>1.5 mg/dl	0-0.5 mg/dl
Erythrocyte Sedimentation Rate	59 mm/hr	2 mm/hr
Influenza A&B	Negative	Negative
COVID-19 PCR	Negative	Negative
Methotrexate Level	<0.02 Umol/L	0.02-5 Umol/L
Cholesterol	221 mg/dl	0-200 mg/dl
Low-density Lipoprotein	>99 mg/dl	0-99 mg/dl

**Figure 1:**
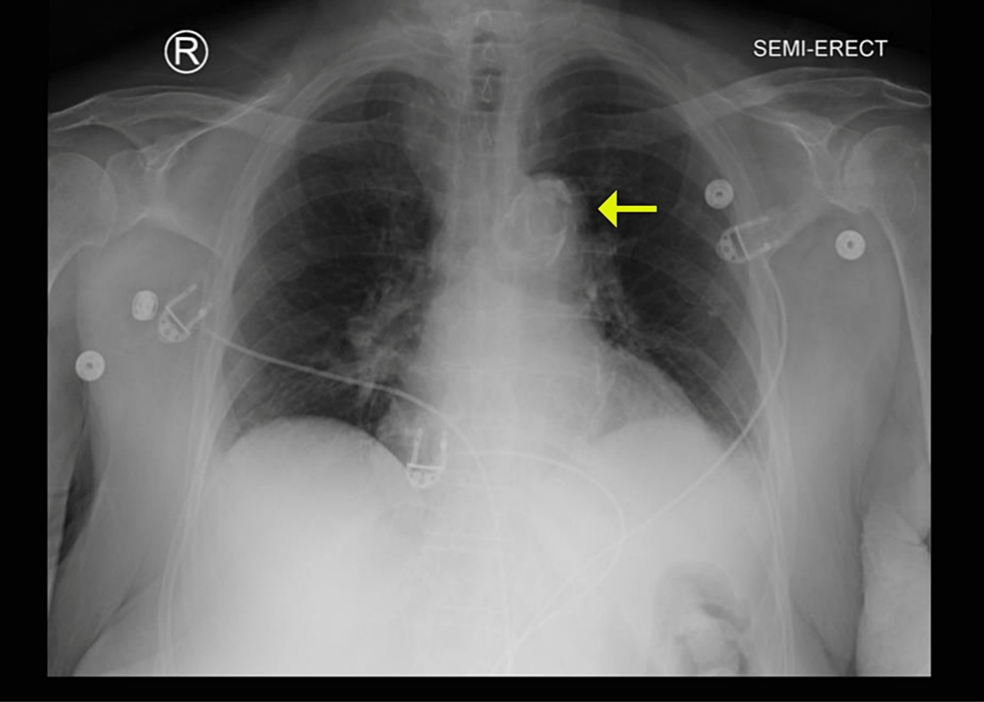
Chest Radiograph Chest X-ray showing unidentified object in the upper mediastinum (arrow).

**Figure 2:**
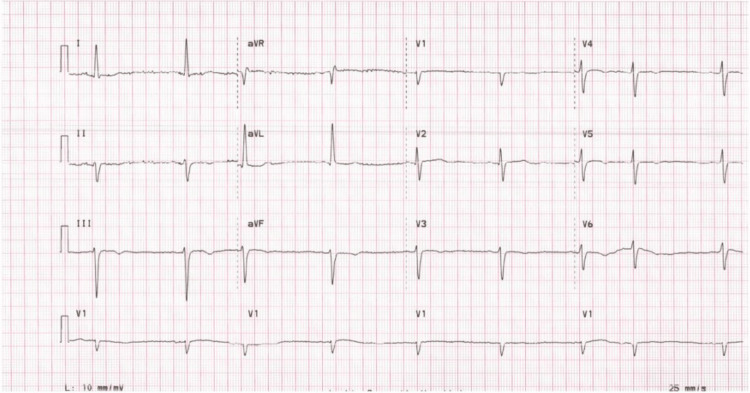
Electrocardiogram Electrocardiogram showing normal sinus rhythm with left anterior hemiblock without significant ST segment or T wave changes.

**Figure 3:**
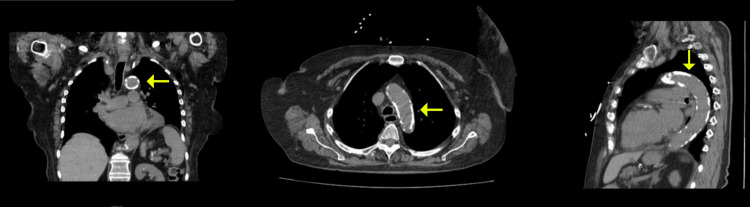
Chest Computed Tomography (CT) Scan Chest CT scan revealed diffuse calcification of the thoracic aorta (arrows).

Chest radiograph showed an unidentified object in the upper mediastinum without evidence of pulmonary infiltrate or edema (Figure 1). Electrocardiography revealed sinus rhythm without ischemic changes (Figure 2). Sputum and blood cultures were obtained, and the patient was admitted to the hospital for further evaluation and treatment. CT scan of the chest was performed and demonstrated severe circumferential calcification of the thoracic aorta with patchy calcification along the vasculature beds (Figure 3). Echocardiogram revealed mild concentric left ventricular (LV) hypertrophy, normal LV segmental wall motion, normal LV ejection fraction (LVEF) of 50%-55%, and normal right ventricle size and systolic function. There was no evidence of valvular heart disease apart from mild mitral annular calcification and aortic valve degenerative calcification without increased pressure gradients.

Blood and sputum cultures resulted without growth and patient’s symptoms were attributed to acute bronchitis. The patient reported improvement in her symptoms and had an uneventful one-day hospital stay. She was educated about her condition, the importance of medication compliance, and outpatient follow-up with her primary care physician (PCP), cardiologist, and rheumatologist. The patient was discharged home after optimizing her cardiovascular risk factors by adding statin, and baby aspirin, along with her home medications for high blood pressure and RA.

## Discussion

Aortic calcification can be incidentally detected in patients undergoing evaluation for cardiopulmonary symptoms or as part of the pre-operative evaluation. Early onset and widespread calcification in multiple vascular beds have been reported in patients with RA [5]. The chronic inflammatory state and immunological vascular damage with RA are the key mechanisms for the accumulation of dystrophic calcification in the blood vessels and soft tissues in this patient population [6]. There has been growing evidence of increased cardiovascular mortality in patients with ascending aortic and/or aortic arch calcification, and furthermore, it is recognized for its perioperative significance of increased complications of cardiac surgeries that requires cross-clamping of the aorta (valve replacement or coronary bypass surgery) [2]. As demonstrated in our case, chest radiographs were the first commonly performed imaging modality that led to the incidental discovery of mediastinal vascular calcification, which was further classified on a thoracic CT scan. While non-invasive CT imaging can allow rapid and effective detection of the prevalence and the severity of vascular calcification, it still is unable to differentiate the pathophysiology of the calcification from being in the intima (atherosclerotic) or in the media (non-atherosclerotic) [2]. However, it was suggested that the calcification pattern and distribution on imaging can give us a clue on the underlying etiology. A patchy calcification pattern is an indicator of atherosclerosis as opposed to a circumferential/continuous pattern, which can indicate underlying inflammatory arteritis or radiation-induced calcification. Certainly, in our case, both types of calcifications were seen with more predominance of the patchy pattern in the abdominal aorta.

It is very important to recognize different risk factors in patients with autoimmune or inflammatory disorders and implement preventive medicine in as they are more prone to CVD which can cause a significant increase in mortality and morbidity.

## Conclusions

In conclusion, RA is associated with an increased predisposition for vascular calcification which can add up to increased morbidity and mortality. Targeting cardiovascular risk factors in this patient population is just as important as disease control. This can be achieved by early recognition of these risk factors and coordinated care between different specialties including PCP, rheumatology, and cardiology.

## References

[1] Yalcinkaya D, Yarlioglues M, Ergun E (2021). Severe cardiovascular involvement in a patient with rheumatoid arthritis. Kardiol Pol.

[2] Abramowitz Y, Jilaihawi H, Chakravarty T, Mack MJ, Makkar RR (2015). Porcelain aorta: a comprehensive review. Circulation.

[3] Turesson C, Jacobsson L, Rydén Ahlgren A, Sturfelt G, Wollmer P, Länne T (2005). Increased stiffness of the abdominal aorta in women with rheumatoid arthritis. Rheumatology (Oxford).

[4] Canpolat U, Gürses KM, Sahiner L, Aksöyek S (2013). Porcelain thoracic aorta in a patient with rheumatoid arthritis. Turk Kardiyol Dern Ars.

[5] Mills R, Alam MH, Alonso-Gonzalez R, Rubens MB, Gatzoulis M (2015). A case of malignant aortic calcification in congenital heart disease and rheumatoid arthritis. Int J Cardiol.

[6] Paccou J, Renard C, Liabeuf S (2014). Coronary and abdominal aorta calcification in rheumatoid arthritis: relationships with traditional cardiovascular risk factors, disease characteristics, and concomitant treatments. J Rheumatol.

